# Modifiable Risk Factors for Dementia in the Indian Context – Where Vascular and Metabolic Ends Meet

**DOI:** 10.1002/alz70858_102465

**Published:** 2025-12-25

**Authors:** Thomas Gregor Issac

**Affiliations:** ^1^ CBR, Bengaluru, karnataka, India

## Abstract

**Background:**

India has the largest population in the world, and ∼153 million people are aged above 60; a figure expected to double by 2050. This demographic shift will likely increase the burden of cognitive impairment and dementia. Strategies such as early identification, identifying vulnerable groups, management of modifiable risk factors, enhancing protective factors and improving cognitive resilience are crucial to prevent or delay the onset of dementia as broadly suggested in literature and by the Lancet Commission's report on dementia prevention, intervention and care.

**Method:**

The Tata Longitudinal Study of Aging (TLSA) and Srinivaspura Aging Neuro Senescence and COGnition (SANSCOG) cohorts are urban and rural cohort studies respectively, based in southern India. The baseline data from these cohorts were used to derive preliminary insights into risk factors for cognitive impairment.

**Result:**

The TLSA and SANSCOG studies have reported 39.84% and 22.77% prevalence of hypertension and diabetes respectively, with the rural population having a greater burden of undiagnosed and untreated conditions placing them at greater risk. Metabolic syndrome affects 46.2% of rural and 54.8% of the urban population. Insulin resistance is particularly high in the urban population (56.5%). The SANSCOG study showed that higher cardiovascular risk was associated with impairment in several cognitive domains.

In addition to factors reported by the Lancet Commission, the cohorts were examined for other potentially modifiable risk factors. High homocysteine levels were found to be associated with impairment in language domain and lesser white matter volume. Poorer sleep duration and efficiency

**Conclusion:**

The results show that the burden of vascular and metabolic risk factors is high in the Indian context and highlight the relationship of these risk factors with cognition in individuals without dementia, suggesting their potential utility as early targets to prevent or delay incidence of dementia. The findings also underscore the need for low‐and middle‐income countries like India, with a high cardiovascular burden, to focus on correcting modifiable risk factors in mid‐life to reduce dementia burden.

